# Frontoparietal cortex and cerebellum contribution to the update of actual and mental motor performance during the day

**DOI:** 10.1038/srep30126

**Published:** 2016-07-22

**Authors:** Laura Bonzano, Luca Roccatagliata, Piero Ruggeri, Charalambos Papaxanthis, Marco Bove

**Affiliations:** 1 Department of Neuroscience, Rehabilitation, Ophthalmology, Genetics, Maternal and Child Health, University of Genoa, Genoa, Italy; 2 Magnetic Resonance Research Centre on Nervous System Diseases, University of Genoa, Genoa, Italy; 3 Department of Health Sciences, University of Genoa, Genoa, Italy; 4 Department of Experimental Medicine, Section of Human Physiology and Centro Polifunzionale di Scienze Motorie, University of Genoa, Genoa, Italy; 5 Université de Bourgogne Franche-Comté, CAPS UMR1093, F-21000 Dijon, France; 6 INSERM, CAPS UMR1093, F-21000 Dijon, France

## Abstract

Actual and imagined movement speed increases from early morning until mid-afternoon. Here, we investigated the neural correlates of these daily changes. Fifteen subjects performed actual and imagined right finger opposition movement sequences at 8 am and 2 pm. Both actual and imagined movements were significantly faster at 2 pm than 8 am. In the morning, actual movements significantly activated the left primary somatosensory and motor areas, and bilaterally the cerebellum; in the afternoon activations were similar but reduced. Contrast analysis revealed greater activity in the cerebellum, the left primary sensorimotor cortex and parietal lobe in the morning than in the afternoon. Imagined movements in the morning significantly activated the parietal association cortices bilaterally, the left supplementary and premotor areas, and the right orbitofrontal cortex and cerebellum. In the afternoon, the frontal lobe was significantly activated with the right cerebellum. Contrast analysis revealed increased activity in the left parietal lobe in the morning than in the afternoon. For both tasks, speed in the morning was significantly related to the BOLD signal in the brain areas resulted more active. These findings suggest that motor performance is continuously updated on a daily basis with a predominant role of the frontoparietal cortex and cerebellum.

An intriguing feature of our motor behavior is the daily modulation of motor performance. Indeed, several motor parameters, such as force^1^,^2^,^3^, speed^4^, reaction time^5^,^6^, and manual dexterity^7^,^8^ progressively improve from early morning until mid-afternoon. Interestingly, it has also been shown that mental movement time, that is the time of an imagined movement, also exhibits daily variations^4^,^9^,^10^. So far, it has been suggested that the daily modulation of physical and mental motor performance follows circadian rhythms, similarly to what occurs for biological mechanisms, such as hormonal secretion, body temperature, and the sleep/awake cycle^11^. Recent findings propose that daily active movements, which also follow the daily rhythm imposed by natural (alternation of dark and light) and social (professional, educational) rules, may be, in addition to other basic circadian factors, a potential factor that regulates the fluctuation of motor performance^12^. The underlying mechanism for the observed daily modulation of physical and mental motor performance would be the continuous update of motor prediction via active movements. Motor prediction is generated by forward models, which are neural processes that mimic the causal flow of the physical process by predicting the future sensorimotor state (e.g., position, velocity) given the efferent copy of the motor command and the current state^13^,^14^. Better performance in the afternoon with respect to the morning, as that observed in the time of mental arm movements^4^, may be due to the continuous update of internal predictive models by means of self-supervised learning^15^, a process that minimizes prediction errors, namely the errors between the output from the forward model and the sensory outcome of the motor command. For instance, it is well established that movement prevention, via arm immobilization, severely affects motor performance^16^,^17^.

Although the daily fluctuation of physical and mental motor performance is well described, the neural substrates of this phenomenon are not yet elucidated.

Here, to investigate these neural substrates, we conducted an fMRI study on a group of fifteen healthy subjects, each participating to two different sessions, in the morning (8 am) and in the afternoon (2 pm). During each session, participants had to actually and mentally perform a simple sequence of finger opposition movements, as accurate and fast as possible. Simultaneous motor performance was measured by using a magnetic-resonance-compatible engineered glove^18^.

The expected performance improvement between the two sessions could be related to changes in the activation patterns of several brain areas. In particular, the hypothesis that this improvement is due to an update of the forward model prediction should be confirmed by changes in the activation patterns of the cerebellum and the parietal cortex. Indeed, the cerebellum has been proposed as the neural site of the updating of state estimation^19^ and the parietal cortex as the neural site of the storage of the updated state estimation^20^. For instance, the demonstration that patients with damage in the posterior parietal cortex are unable to perform motor imagery tasks^21^, and thus to generate internal predictions of their own movements, supports this hypothesis.

Further, actual and mental performance of sequential finger movements has been found to share a common activity in the widely distributed frontoparietal network^22^,^23^. Since frontoparietal cortex might play a significant role in storing and accessing motor representation, we also hypothesized significant changes in the activity of this network related to the improvement of actual and motor performance throughout the day.

## Material and methods

### Participants

This study was reviewed and approved by the local ethics committee (Comitato Etico Regione Liguria, IRCCS Azienda Ospedaliera Universitaria San Martino – IST, Genoa, Italy), and a written informed consent was obtained from all subjects. The study was carried out in accordance with the approved guidelines.

Fifteen healthy volunteers were recruited for this study (7 females and 8 males, mean age = 23.4 ± 2.6 years). None of the enrolled participants presented with a history of neurological or psychiatric disorders or use of psychoactive drugs. All had a similar normal routine of daytime activity (6–7 am to 11–12 pm) alternating with a nighttime sleep. Their chronotype (Morningness-Eveningness Questionnaire^24^) was moderate morning type (n = 4) or neither type (n = 11). Participants were right handed according to a modified Italian-translated Edinburgh Handedness Inventory^25^ and naive to the specific purpose of the study. To evaluate the participants’ motor imagery ability, an Italian adaptation of the Motor Imagery Questionnaire - Revised (MIQR)^26^ was adopted. This questionnaire measures the difficulty (from 1 = very easy to 7 = very difficult) experienced by the participant in performing visual and kinesthetic imagery with a 7-point scale based on 8 questions (score from 8-best to 56-worst). During visual imagery, a subject visualizes herself/himself performing a specific task with a third-person perspective, while during kinesthetic imagery a subject feels herself/himself performing a specific task with a first-person perspective. Participants showed very good imagery ability (mean total score = 16.2 ± 6.3; mean visual score = 7.9 ± 3.2, mean kinesthetic score = 8.3 ± 3.3).

### Neural correlates of actual and imagined finger movements

By using fMRI, we investigated brain activity during actual and imagined finger movements in two sessions at two different times of the same day: at 8 am (AM session) and at 2 pm (PM session). These sessions were chosen on the basis of a preliminary experiment in which 10 healthy subjects performed the same tasks at four different times of the day (8 am, 11 am, 2 pm and 5 pm) to test performance fluctuations during the day; the minimum performance level (i.e., longest task duration) for both conditions was found at 8 am whilst the maximum (i.e., shortest task duration) in the early afternoon (data not shown). Two days before the MRI scan, participants had a one-hour practice session, where they were given an overview of MRI procedures, were tested for imagery abilities, and familiarized themselves with the glove-device and the required tasks.

During each session, participants were asked to actually perform (physical execution - PE condition) and to mentally simulate (first-person perspective motor imagery - MI condition) a simple sequence of finger opposition movements with their right hand (thumb-to-index, medium, ring and little finger) as accurate and fast as possible. The order of actual and imagined movements was randomly assigned for each participant and session. Within each fMRI run, participants had to either perform the described finger motor task (task) or to stay at rest without making any overt movement (rest), according to a boxcar design with two 30-s task blocks alternating with two 30-s rest blocks. For each participant, we acquired 2 fMRI runs per condition. Participants were asked to keep their eyes closed during the whole experimental session, to avoid possible confounding effects due to the integration of sensorimotor and visual information during the task and to avoid visual cortical activation.

We were able to quantify both actual and mental performance concomitantly with fMRI acquisition by an ad hoc developed MR-compatible engineered glove^18^,^27^. Specifically, actual movements consisted in 5 repetitions of the sequence (thumb-to-index, medium, ring and little finger) and motor performance was assessed by the whole movement duration. Accuracy was assessed by means of the *ad hoc* developed software controlling the glove system: the required sequence of finger movements was pre-set when implementing the experimental protocol, and the number of errors was calculated on the basis of the comparison between the “desired” sequence and the sequence “actually performed” by the subject.

For mental movements, the participants were instructed to tap the index with the thumb at the beginning and the end of the mental task; thus, we also evaluated mental performance via the whole mental movement duration. In both conditions, participants had to repeat the task until the end of the block.

Furthermore, to assess if a counterbalanced design would affect the performance of actual and mental movements, we run a control experiment. In details, 15 new participants (8 females and 7 males, mean age 23.7 ± 2.3 years) carried out 2 tests of imagined movements during 2 days (1^st^ day at 2 pm and 2^nd^ day at 8 am) and, one week later, 2 tests of actual movements during 2 days (1^st^ day at 2 pm and 2^nd^ day at 8 am). The motor task was the same as the one used in the main experiment, but tested in the opposite order (afternoon session followed by morning session).

### MRI acquisition

Each participant underwent two brain MRI examinations on the same day (AM and PM sessions), with structural and fMRI sequences performed on a 1.5 T MR system (Signa Excite HDxt, General Electric Healthcare,WI, USA). All the series covered the whole brain; the MRI protocol included an axial FLAIR sequence (slice thickness = 5 mm; TR = 9002 ms; TE = 97.5 ms; inversion time = 2250 ms; flip angle = 90°; FOV = 240 × 240 mm; matrix = 512 × 512) to exclude incidental findings in the enrolled subjects (AM session only), and an axial T2 sequence (slice thickness = 5 mm; TR = 6300 ms; TE = 123.7 ms; FOV = 260 × 260 mm; matrix = 256 × 256) used as structural reference for the fMRI acquisition. Functional MRI data were acquired with axial T2*-weighted single-shot spin-echo echoplanar sequences (slice thickness = 5 mm; TR = 3000 ms; TE = 40 ms; FOV = 260 × 260 mm; matrix = 64 × 64). Each fMRI sequence lasted 2:09 min; the first three brain volumes (i.e., 9 s) were discarded to allow steady-state magnetization.

### fMRI pre-processing

SPM12 software (Wellcome Department of Imaging Neuroscience, London, UK) was used for fMRI processing and statistical analysis, as described elsewhere^28^. For each participant, the first image was used as a reference to which all the subsequent scans were realigned, and the 6 parameters describing the rigid body transformation between each source image and the reference image were used to re-sample each image to apply motion correction. Then, slice timing was applied to minimize timing-errors between slices and the functional images were normalized to the Montreal Neurological Institute (MNI) template brain image using a 12-parameter affine transformation, re-sampled to 2 × 2 × 2 mm^3^ voxels and smoothed with an 8 mm full-width at half-maximum isotropic Gaussian kernel to increase the signal-to-noise ratio.

### Behavioral data analysis

Participants wore a sensor-engineered glove on their right hand. Data were acquired at 1 KHz by means of a data acquisition board (USB-1208FS, Measurement Computing, USA); an ad hoc software tool recorded the occurrence of each finger touch in the motor sequence^29^. The task duration for actual and mental movements, as well as the number of errors for actual movements, were computed considering the five repetitions of the proposed sequence (actual or mental). Mean values and standard errors were calculated for each participant.

### Statistical analysis

We checked that all kinematics variables were normally distributed (p > 0.05, Shapiro-Wilk W test). To test changes in the actual and mental performance through the day, we performed a two-way repeated-measures analysis of variance (ANOVA) with *time-of-day* (AM and PM) and *task* (PE and MI) as independent within-subject factors. Paired Student’s t-test was used to assess differences in the number of errors during actual movements between the AM and PM sessions.

To test possible differences between the main and the control experiment, we performed a repeated-measures ANOVA with time-of-day (AM and PM), task (PE and MI) and order (AM1st and PM1st) as within-subject factors. Factor order indicated which was the first session tested (AM was the first session in the main experiment, PM was the first session in the control experiment).

For the fMRI analysis, a general linear model was used to identify the voxels with task-related signal changes at the individual level. Task-related *t* contrast images were created for every participant in each experimental condition (PE-AM, PE-PM, MI-AM, MI-PM). These first-level models were then introduced into a second-level random-effect analysis to allow for population inferences by an ANOVA model including the different experimental conditions. For the one-sample t-tests to obtain the group activation maps in each condition, a height threshold of p < 0.05 FWE-corrected was applied. Extent threshold was arbitrarily set at k = 50 voxels. The analysis of statistical contrasts between experimental sessions for each condition was conducted with height threshold of p < 0.001 uncorrected and minimum cluster size of 25 voxels.

In addition, on the basis of the contrast analysis results, to investigate the influence of the frontoparietal cortex and cerebellum on task performance, the first eigenvariate of the BOLD signal was extracted in the corresponding clusters in the group activation maps obtained in the AM session. Then, the relationship between the extracted values and task duration was assessed, respectively, for the PE and for the MI condition.

Data in the text are reported as means ± SD.

## Results

### Behavioral data

**Figure 1:**
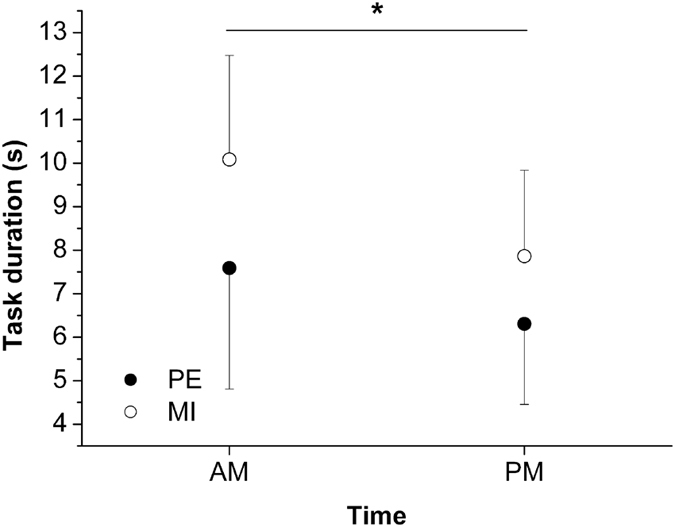
Durations of the two task conditions (PE = physical execution, MI = motor imagery) in the two sessions (AM = 8 am, PM = 2 pm). The error bars represent the SD. *Indicates significant difference at 2 pm compared to the respective value at 8 am.

As shown in Fig. 1, both actual and imagined movements were significantly slower at 8 am than at 2 pm (main effect of *time-of-day*: F(1, 28) = 25.46, p < 0.0001; task duration in the PE condition: 7.59 ± 2.78 s and 6.31 ± 1.85 s, respectively; MI: 10.08 ± 2.39 s and 7.86 ± 1.97 s, respectively). Furthermore, imagined movement durations were longer than actual movement durations in both experimental sessions (main effect of *task*: F(1, 28) = 7.18, p = 0.01). Although imagined movement duration decreased more than actual movement duration from 8 am to 2 pm, there was no significant *time-of-day* × *task* interaction (F(1, 28) = 1.82, p = 0.19).

No significant difference between the two experimental sessions was found in the number of errors for actual movements (mean number of errors AM: 0.13 ± 0.35, PM: 0.2 ± 0.41; t = 0.43, p = 0.67).

The analysis in the control experiment showed similar results as in the main protocol, namely a decrease in movement duration during the day. Precisely, movement duration was 7.71 ± 1.11 s at 8 am and 6.38 ± 0.87 s at 2 pm for actual movements and 9.59 ± 1.49 s at 8 am and 7.77 ± 1.15 s at 2 pm for imagined movements. Considering the main and the control experiments together no effect of order was found (F(1, 56) = 0.05, p = 0.82), indicating that the reduction of task duration in the afternoon was not related to the first session of performance during the day. Further, the lack of *time-of-day* × *task* × *order* interaction (F(1, 56) = 0.35, p = 0.56) pointed out the same trends between the main and the control experiments.

### Brain activation during actual movements

**Figure 2:**
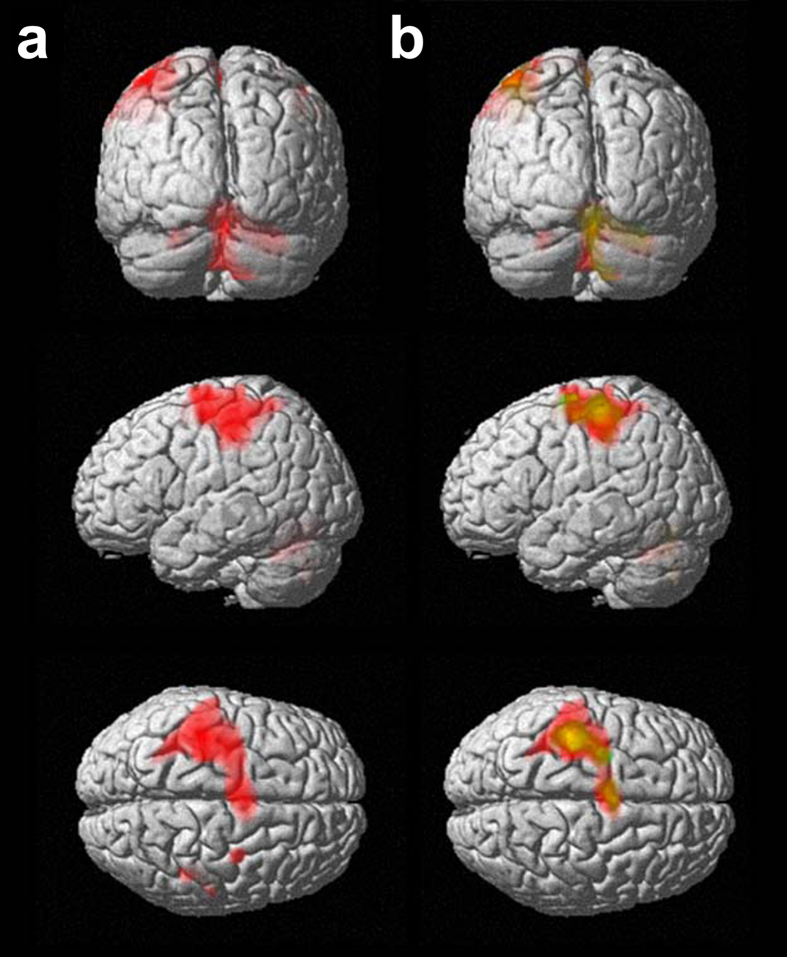
Group activation blobs displayed on a rendering surface for the physical execution (PE) condition (height threshold p < 0.05 FWE-corrected; extent threshold k = 50 voxels): (**a**) morning (AM) and afternoon (PM) sessions pooled together (see Table 1 for details), (**b**) AM in red, PM in green (see Table 2 for details). Yellow represents the superposition of the blobs obtained in the two sessions.

For the PE condition, considering the AM and PM sessions together, significant group activations were observed in the left sensorimotor areas and bilaterally in the cerebellum (Fig. 2a and Table 1).

Figure 2b and Table 2 depict brain activation patterns for the AM and PM sessions, separately. In the PE-AM condition, we found significant activation peaks in the left primary somatosensory cortex (S1), supplementary motor area (SMA), and premotor cortex (PMC). The cerebellum was bilaterally activated, with peaks in the right Lobule IV-V, Vermis_6, and Vermis_8, and left Lobule VI. In particular, as it can be appreciated in Fig. 2b, the cortical cluster covered part of the precentral and postcentral gyri. The performance of actual movements in the afternoon (PE-PM condition) elicited reduced activations in the same cortical areas (left S1, SMA and PMC) and in the cerebellum (only the right cerebellar hemisphere was active: Lobule IV-V, Lobule VI, and Vermis_6).

The statistical contrast analysis PM > AM did not show suprathreshold clusters, thus confirming greater brain activation in the morning with respect to the afternoon. On the other hand, the AM > PM contrast revealed greater activation in the cerebellum (left Lobule IV-V and right Crus I), in the left Brodmann area (BA) 1 and BA 40 (Table 3).

### Brain activation during mental movements

**Figure 3:**
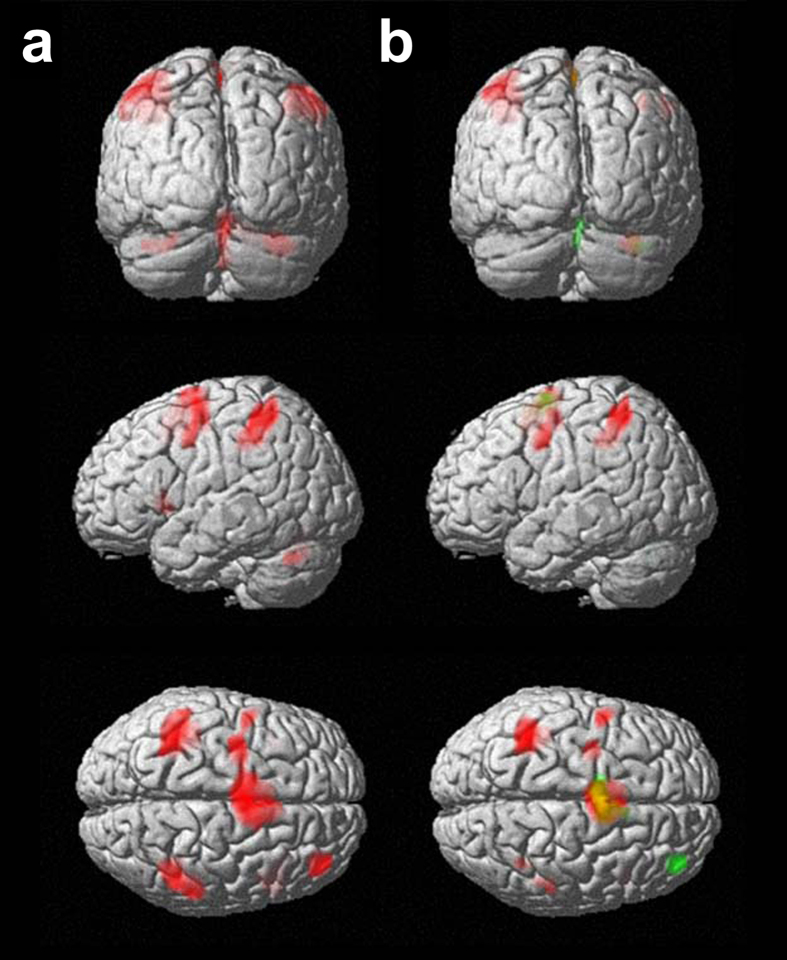
Group activation blobs displayed on a rendering surface for the motor imagery (MI) condition (height threshold p < 0.05 FWE-corrected; extent threshold k = 50 voxels): (**a**) morning (AM) and afternoon (PM) sessions pooled together (see Table 1 for details), (**b**) AM in red, PM in green (see Table 2 for details). Yellow represents the superposition of the blobs obtained in the two sessions.

Considering the AM and PM sessions together, the group activation maps obtained during imagined movements included significant clusters bilaterally in the supplementary motor areas, the prefrontal cortex, the parietal cortex, and the cerebellum (Fig. 3a and Table 1).

Brain activations during the MI condition resulted to be different between the AM and PM sessions (see Fig. 3b and Table 2). Indeed, in the AM session, significant peaks of activation were found in the left and right parietal association cortices (BA 40), the left SMA and PMC, the right orbitofrontal cortex (BA 47), and the right Lobule VI of the cerebellum. The cluster in the left parietal lobe covered part of the inferior and superior parietal lobules, including suprathreshold voxels in the left BA 40, BA 5 and BA 7. In the PM session, peaks of activation were found bilaterally in the SMA and the right rostral prefrontal cortex (BA 10). The right cerebellum was also active, including more clusters (Lobule IV-V, Lobule VI, Vermis_7, and Crus I).

As for actual movements (PE task), the PM > AM statistical contrast analysis did not show suprathreshold clusters. On the contrary, the AM > PM contrast revealed increased activity in the left parietal lobe (BA 7) (Table 3).

### Correlations of BOLD signal with actual and mental movement durations

**Figure 4:**
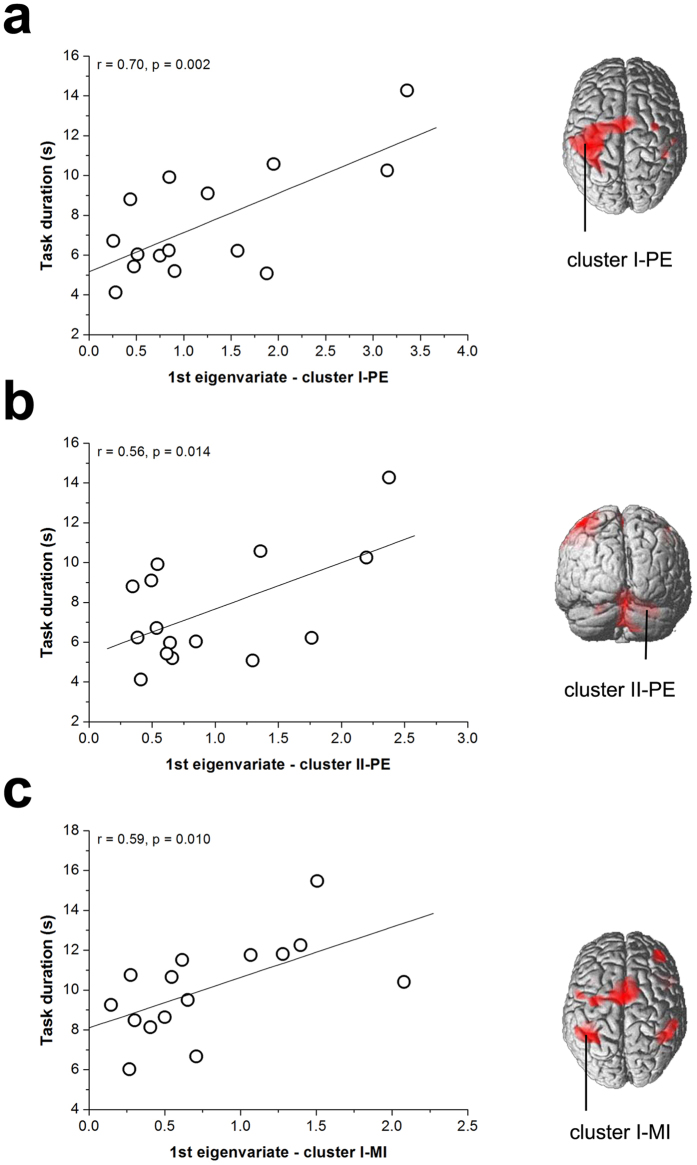
Linear relationships during the morning session between task duration and the 1^st^ eigenvariate of the BOLD signal in: (**a**) cluster I–PE, (**b**) cluster II–PE, (**c**) cluster I–MI.

On the basis of the contrast analysis, the first eigenvariate of the BOLD signal was extracted in the corresponding clusters in the group activation maps obtained in the AM session (i.e., the cluster located in the left frontoparietal areas in the PE-AM condition – “cluster I–PE”, the cluster located in the right cerebellum in the PE-AM condition – “cluster II–PE”, and the cluster located in the left parietal lobe in the MI-AM condition – “cluster I–MI”). Figure 4 shows the linear relationships between the extracted value and task duration for each participant in the PE and in the MI conditions, respectively. All the correlations resulted to be direct and statistically significant (cluster I–PE: r = 0.70, p = 0.002; cluster II–PE: r = 0.56, p = 0.014; cluster I–MI: r = 0.59, p = 0.010). These results indicate that, in both actual and mental tasks, the subjects performing the task with longer durations were those showing more activity in the clusters including the areas resulted significantly activated in the morning with respect to the afternoon.

## Discussion

In this work we investigated the neural correlates of movement execution and imagery improvement during the day. Fifteen healthy subjects, participating in two different fMRI sessions (8 AM and 2 PM), were asked to actually and mentally perform a simple sequence of finger opposition movements as accurate and fast as possible. We found that actual and imagined movements were significantly faster at 2 pm than at 8 am. These results, in agreement with previous studies^4^,^9^, suggest that motor performance shows substantial changes during the day. It should be noted that although temporal discrepancies between actual and imagined movements decreased from 8 am to 2 pm, they still remained significantly different at 2 pm. These findings are in contrast with those of previous studies that reported isochrony between actual and imagined movements at 2 pm^4^,^12^. Such differences could be explained by the motor task and the body-parts (arm pointing vs. finger tapping) involved in the movement, as well as by the fact that in the previous studies participants carried out actual and imagined movement every 3 h from 8 am, while in the current study participants carried out actual and imagined movements at 8 am and 2 pm only.

Movement execution engaged similar brain activation maps during the AM and PM sessions, mainly including significant clusters in the left sensorimotor areas and in the cerebellum, with a reduction of activation in the PM session compared with the AM session. Indeed, in the AM session the statistical contrast analysis revealed greater activation in the cerebellum (left Lobule IV-V and right Crus I), and in the left primary sensorimotor cortex (BA 1) and parietal lobe (BA 40). On the contrary, motor imagery induced a different distribution of brain activations between the AM and PM sessions. Particularly, in the AM session significant peaks of activation were found in the left and right parietal cortices, the left SMA and PMC, the right orbitofrontal cortex, and the right Lobule VI of the cerebellum. In the PM session, activation peaks were found in the SMA bilaterally and in the right prefrontal cortex. The right cerebellum was also active, including clusters in the Lobule IV-V, Lobule VI, Vermis_7, and Crus I. Specifically, the contrast analysis revealed increased activation in the left parietal lobe (BA 7) in the morning with respect to the afternoon, suggesting that kinesthetic representations stored in the parietal cortex must be first activated and organized (e.g., in the morning) to entrain other brain regions that are active (e.g., in the afternoon) during movement simulation^21^.

These findings suggest that the left parietal area plays a main role in controlling the performance of both actual and mental movements in the morning. In particular, the left primary somatosensory cortex and inferior parietal lobe were more active in the morning than in the afternoon during actual movements, whilst the superior parietal lobule showed increased activation in the morning in the mental movement condition. The superior and inferior parietal lobules, together with the cerebellum, are involved in the feedforward control of movements^20^,^30^,^31^,^32^,^33^,^34^. Also, posterior parietal brain activation within the superior parietal lobe is linked to various working memory-related sub-processes, as storage of information^35^ and mental representation of space and time^36^. In a seminal study, Sirigu *et al.* showed that patients with lesions restricted to the parietal cortex were found to be impaired selectively at predicting, through mental imagery, the time necessary to perform differentiated finger movements and visually-guided pointing gestures, in comparison to normal individuals. They concluded that the posterior parietal areas are important for the mental representation of movements, and more precisely for accurate estimation of the time required to complete them.

Furthermore, for both actual and mental movements, we also observed that task duration in the morning session was significantly related to the BOLD signal in the activation clusters including the brain areas that resulted to be more active in the morning than in the afternoon.

Recently, Picard *et al.*^37^ measured metabolic and neuron activity in the primary motor cortex of monkeys who had extensive training (1–6 years) on sequential movement tasks. They found, after extended practice, a widespread alteration in the relationship between metabolic activity and neuron activity associated with practice on a skilled sequence of movements. This decoupling of metabolic and neuron activity implies that practice leading to skilled performance results in more efficient generation of neuronal activity in primary motor cortex. In our case, we think that a reduction in activation accompanying an improvement in performance could be related to improved efficiency of the recruited neural circuits.

Notably, although motor imagery and physical execution showed some difference in clusters activation^38^,^39^,^40^, the pattern of activation in the inferior and superior parietal lobules, as well as the motor-related regions including the premotor and supplementary cortex and the cerebellum, is similar for actual and mental movements, as also shown in several neuroimaging experiments that investigated sequential finger-to-thumb opposition tasks^40^,^41^,^42^,^43^,^44^,^45^,^46^. On these bases, we could assume that parietal frontal areas play an important role in updating daily sensorimotor experience to build a vivid representation of our movement. In our case, the larger the duration of the motor task (i.e., lower motor performance), the higher the activity in these areas in the morning. Recent neuroimaging studies have also demonstrated that the parietal lobe is involved in detecting self-generated movements^39^,^47^,^48^,^49^. Therefore, the strong activity observed in the parietal cortex in the morning can be considered as the activation signal of a cortico-cortical processing loop necessary to update the predictive internal model. When the internal model is precise enough, thanks to the active movements/experience during the day, the activity in these brain areas can be reduced, as observed in the PM session in our study.

In this context, it has been shown that the failure of incorporation of the rubber hand induced an increase of activity in the parietal cortex^50^. In particular, a negative correlation was found between the proprioceptive measure of the rubber hand illusion and the regional cerebral blood flow in the contralateral parietal cortex. Although the rubber hand illusion is an experimental paradigm that isolates the pure sense of body ownership in the absence of movement and efferent information, and it shares some electrophysiological brain mechanisms in the frontoparietal cortex with motor imagery (i.e., the mental rehearsal of movements in the absence of execution and thus sensory feedback)^51^.

Our study only investigated performance and activation changes during task performed with the right (dominant) hand, in accordance to previous behavioral results. This could be a potential hint for future work considering a left-hand condition, which would show whether activation associated with time of the day is consistently on the left or right hemisphere or contralateral to the hand moving.

All these findings might suggest that even if when I decide to write or to kinesthetically imagine to write I do not need to look for my hand for the simple reason that my hand is “always there” present with me^52^, the neural process allowing me to feel that “my body” belongs to me, and ever present in my mental life, (i.e., body ownership)^53^ needs a higher level of mental resources in the morning with respect to the afternoon. This can be explained through the need to recall my sensorimotor experience, that is not always available at the beginning of the day and that can be improved by means of active movements/experience during the day.

## Conclusions

We found that the duration of a finger motor task significantly decreased in the afternoon with respect to the morning for both actual and imagined movement performance, as effect of daily activity^12^. These differences were accompanied by a reduction of functional activation in the frontoparietal cortex and cerebellum, supporting the role of these areas in updating motor performance on a daily basis. Although a general effect of time of day in brain activation has been reported in previous studies^54^,^55^,^56^,^57^ and thus must be taken into account as a cofactor in neuroimaging analysis (for instance, cerebral blood flow velocity, an indicator of cortical activity, is also time of day dependent with maximum at 00:00 h^56^), the effect of time of day in brain activation patterns in actual and mental movements observed here suggest plastic changes in the brain circuits due to the daily update of the predictive internal model.

Therefore, the effect of the course of the day becomes a crucial factor that has to be taken into account when planning experiments based on the performance of actual or mental motor tasks. In addition, this aspect could have important implications especially for patients affected by neurological diseases with specific brain structural and/or functional alterations that could differently affect motor performance depending on the time of the testing session. Indeed, data obtained in different times of the day might lead to biased interpretations of results and erroneous assessment of disease severity.

Finally, a preliminary analysis of the motor performance and brain activity during the day could help design a proper rehabilitation protocol.

## Additional Information

**How to cite this article**: Bonzano, L. *et al.* Frontoparietal cortex and cerebellum contribution to the update of actual and mental motor performance during the day. *Sci. Rep.* **6**, 30126; doi: 10.1038/srep30126 (2016).

## Figures and Tables

**Table 1:** Coordinates of peak activations for the physical execution (PE) and the motor imagery (MI) conditions (morning - AM and afternoon - PM sessions pooled together) (height threshold p < 0.05 FWE-corrected; extent threshold k = 50 voxels).

Condition	Cluster Size	Voxel T	MNI Coordinate: x y z (mm)	Hemisphere	Anatomical area
PE	3534	11.43	2 −66 −20	R	Posterior Cerebellum (Vermis_6)
		10.68	16 −52 −22	R	Anterior Cerebellum (Lobule IV-V)
		8.87	6 −70 −38	R	Posterior Cerebellum (Vermis_8)
	4914	10.64	−38 −26 64	L	Precentral Gyrus (BA 4)
		8.83	−6 −4 64	L	Medial Frontal Gyrus (BA 6)
		7.32	−34 −12 70	L	Precentral Gyrus (BA 6)
	98	6.36	34 −6 66	R	Middle Frontal Gyrus (BA 6)
	175	5.75	48 −38 54	R	Inferior Parietal Lobule (BA 40)
		5.6	44 −34 46	R	Inferior Parietal Lobule (BA 40)
		5.55	56 −24 46	R	Postcentral Gyrus (BA 2)
MI	2526	9.74	−4 −4 66	L	Medial Frontal Gyrus (BA 6)
		9.14	8 0 66	R	Medial Frontal Gyrus (BA 6)
		7.26	−32 −6 58	L	Middle Frontal Gyrus (BA 6)
	1047	8.98	34 −62 −30	R	Posterior Cerebellum (Lobule VI)
		7.06	2 −66 −26	R	Posterior Cerebellum (Vermis_7)
		6.47	16 −50 −24	R	Anterior Cerebellum (Lobule IV-V)
	322	7.85	40 46 30	R	Middle Frontal Gyrus (BA 10)
	1194	6.89	−42 −34 42	L	Inferior Parietal Lobule (BA 40)
		6.64	−34 −46 52	L	Inferior Parietal Lobule (BA 40)
		6.52	−44 −46 64	L	Inferior Parietal Lobule (BA 40)
	675	6.85	48 14 −4	R	Superior Temporal Gyrus (BA 22)
		6.07	36 20 4	R	Insula (BA 13)
		5.74	50 10 12	R	Precentral Gyrus (BA 44)
	745	6.74	54 −34 48	R	Inferior Parietal Lobule (BA 40)
		6.2	42 −48 50	R	Inferior Parietal Lobule (BA 40)
	217	6.43	−26 −72 −26	L	Posterior Cerebellum (Crus I)
		6.33	−32 −62 −32	L	Posterior Cerebellum (Crus I)
		6.13	−40 −62 −30	L	Posterior Cerebellum (Crus I)
	140	5.91	−34 16 8	L	Insula (BA 13)
		5.48	−42 12 4	L	Insula (BA 13)

**Table 2:** Coordinates of peak activations for the physical execution (PE) and the motor imagery (MI) conditions (morning - AM and afternoon - PM sessions in comparison) (height threshold p < 0.05 FWE-corrected; extent threshold k = 50 voxels).

Anatomical area	Hemisphere	AM				PM			
		PE		MI		PE		MI	
		MNI Coordinate: x y z (mm)	voxel T	MNI Coordinate: x y z (mm)	voxel T	MNI Coordinate: x y z (mm)	voxel T	MNI Coordinate: x y z (mm)	voxel T
*Parietal lobe*									
Primary somatosensory cortex (Postcentral Gyrus, BA 3)	L	−40 −26 64	9.79			−40 −26 66	7.53		
Association cortex (Inferior Parietal Lobule, BA 40)	L			−34 −46 52	6.46				
	L			−42 −48 62	6.42				
	L			−42 −36 42	5.95				
	R			38 −52 48	5.71				
	R			54 −34 48	5.68				
*Frontal lobe*									
Supplementary motor area (Medial Frontal Gyrus, BA 6)	L	−6 −4 62	7.65	0 −2 66	8.21	−6 −4 64	6.8	−6 −4 64	7.42
Premotor cortex (Superior Frontal Gyrus, BA 6)	L	−14 −8 74	7.15						
Premotor cortex (Middle Frontal Gyrus, BA 6)	L			−54 0 46	7.28	−28 −8 66	5.74		
	L			−32 −6 58	6.4				
Orbitofrontal cortex (Inferior Frontal Gyrus, BA 47)	R			46 14 −2	5.64				
Supplementary motor area (Medial Frontal Gyrus, BA 6)	R							8 0 66	6.84
	R							8 14 50	5.61
Rostral prefrontal cortex (Middle Frontal Gyrus, BA 10)	R							38 46 28	7.03
*Cerebellum*									
Anterior (Lobule IV–V)	R	16 −52 −22	9.42			16 −52 −20	8.06	14 −50 −22	5.42
Posterior (Lobule VI)	R			34 −62 −30	8.02	30 −62 −24	6.89	30 −66 −24	5.22
Posterior (Vermis_6)	R	2 −66 −20	9.91			2 −66 −20	8.7		
Posterior (Vermis_7)	R							2 −66 −26	6.19
Posterior (Vermis_8)	R	4 −70 −36	7.85						
Posterior (Crus I)	R							36 −62 −30	6.51
Anterior (Lobule VI)	L	−26 −58 −28	6.91						
Posterior (Lobule VI)	L	−26 −66 −26	6.3						

**Table 3:** Coordinates of peak activations for the comparisons between AM and PM sessions.

Contrast	Anatomical area	Hemisphere	MNI Coordinate: x y z (mm)	voxel T
PE-AM > PE-PM	*Parietal Lobe*			
	Primary somatosensory cortex (Postcentral Gyrus, BA 1)	L	−48 −28 62	3.46
	Association cortex (Inferior Parietal Lobule, BA 40)	L	−58 −32 50	3.44
	*Cerebellum*			
	Anterior (Lobule IV–V)	L	−4 −44 −26	4.21
	Posterior (Crus I)	R	44 −64 −24	4.06
PE-PM > PE-AM	no suprathreshold clusters			
MI-AM > MI-PM	*Parietal Lobe*			
	Association cortex (Superior Parietal Lobule, BA 7)	L	−30 −72 44	3.53
MI-PM > MI-AM	no suprathreshold clusters			

Differences in activation were considered significant when reaching p < 0.001, k > 25 voxels.
